# Promoting Male Involvement in Family Planning: Insights From the No-Scalpel Vasectomy Program of Davao City, Philippines

**DOI:** 10.9745/GHSP-D-24-00229

**Published:** 2024-10-29

**Authors:** June Harvey Flores, Vergil de Claro, Tomas Miguel Ababon, Jerrielyn Lewis, Lady Jedfeliz Molleno, Laurentiu Stan

**Affiliations:** a RTI International Philippines, Pasig City, Philippines.; b Faculty of Medicine and Pharmacy, Vrije Universiteit Brussel, Brussels, Belgium.; c City Health Office, Davao City, Philippines.

## Abstract

Despite global consensus on the importance of male involvement in family planning, disparities persist in low- and middle-income countries, where women continue to bear the responsibility for these initiatives. The Philippines, with a high fertility rate and unmet family planning needs, exemplifies this challenge. We present the experiences and lessons learned from implementing the no-scalpel vasectomy (NSV) program in Davao City, showcasing its potential for increasing male engagement in family planning decisions. Launched in 2008, the program aimed to address gender disparity by promoting NSV as a safe and effective contraceptive alternative to female-centric methods. Through the use of culturally sensitive information campaigns and couple-focused counseling, the program challenged traditional notions of masculinity and encouraged shared decision-making. Strong local government commitment and public-private partnerships played key roles in driving the program’s success. Results showed an average annual increase of 80% in NSV clients over the past 3 years compared to before the COVID-19 pandemic, underscoring its effectiveness. The program presents a compelling intervention model for similar initiatives, highlighting how overcoming cultural barriers, infrastructure limitations, and budgetary constraints through policy advocacy, strategic partnerships, and tailored approaches can significantly boost male involvement in family planning and improve reproductive health outcomes within communities.

## Abstract (precis)

The no-scalpel vasectomy program in Davao City, Philippines, serves as a promising model for engaging men in family planning and addressing gender disparities in such initiatives.

## Teaser

**Key Findings:**

- The no-scalpel vasectomy (NSV) program of Davao City, Philippines, saw an 80% increase in procedures over the last 3 years compared to its pre-pandemic annual average, highlighting a growing acceptance of men as family planning users.
- Men opting for NSV are showing a more positive attitude toward family planning, seeing it as no longer just a woman’s concern but a shared responsibility.
- Addressing cultural barriers, socioeconomic limitations, and health care access issues is necessary to promote inclusivity and participation of men in family planning initiatives.

**Key Implications:**

- The success of the Davao City NSV program in training health care workers from other regions highlights the pressing need for a nationally standardized network of training hubs that should focus on skills development, counseling and communication, advocacy and community engagement, and more effective male participation in family planning efforts.
- The program demonstrates that informed men become strong advocates within their communities, influencing peers toward positive family planning choices. Program managers should prioritize targeted educational campaigns, informing men about family planning options and their role in shared decision-making with their partners.

## INTRODUCTION

In many low- and middle-income countries, there has been a disproportionate emphasis on women in family planning initiatives. Despite global commitments and international consensus highlighting the importance of greater male involvement, this has not translated into encouraging results.^1^ Traditional gender roles in many cultures position men as the primary decision-makers regarding family planning, often placing the burden of the physical and psychological effects of chosen methods squarely on women. Men’s decisions may prioritize methods that impact women directly or focus on financial considerations and societal expectations, often overlooking the broader implications for women’s health and well-being. This imbalance in shared responsibility weakens the effectiveness of family planning programs, which historically have centered on women while neglecting men as active participants and clients, thus limiting their engagement primarily to financial support.^2^^–^^4^

The shifting societal norms and evolving perspectives on gender equality have underscored the importance of engaging men in family planning efforts, contributing to the growing recognition of male involvement as a crucial component for successful reproductive health programs either as users, supportive partners, or agents of change.^5^^,^^6^ In 1994, the Programme of Action adopted at the International Conference of Population and Development formally acknowledged the importance of men’s involvement and advocated for a comprehensive approach to sexual and reproductive health.^7^

One of the key factors contributing to the low involvement of men in family planning is the limited number of contraceptive options available to them compared to women. Hence, the introduction of the no-scalpel vasectomy (NSV) method represented a significant advancement in family planning, particularly for male participation. NSV offers a safe, effective, and permanent contraceptive option for men, providing an alternative to female-centric methods, such as oral contraceptives, intrauterine devices, and tubal ligation.^8^ Its noninvasive nature and quick recovery time make it an appealing choice for men seeking a more active role in family planning decisions.^9^^,^^10^ Additionally, promoting NSV with gender-transformative interventions and couples counseling aligns with the goal of gender equity and shared responsibility in reproductive health. Expanding contraceptive options empowers couples to make informed choices about family size and spacing, ultimately improving reproductive health outcomes.

The introduction of the NSV method represented a significant advancement in family planning, particularly for male participation.

However, despite ongoing family planning initiatives in the Philippines, the objectives of reducing high fertility rates and meeting FP needs remain elusive, particularly among marginalized communities.^11^^,^^12^ While men’s involvement has been acknowledged and advocated for in national policies,^13^^,^^14^ FP interventions still predominantly focus on women’s contraceptive use. Furthermore, cultural, religious, and socioeconomic factors have continuously influenced the utilization of reproductive health services in the country, highlighting the need for innovative approaches to comprehensively address these barriers.^15^

In this report, we present the challenges encountered and actions taken during the implementation of the Family Planning for Men Program Through No-Scalpel Vasectomy in Davao City, Philippines, to learn from and inform future initiatives aimed at fostering male participation in family planning programs.

## FAMILY PLANNING FOR MEN PROGRAM IN DAVAO CITY

Davao City, a bustling metropolis in Southern Philippines, ranks as one of the most populous cities in the country. With a population of 1.78 million in 2020, it stands as the center of trade, commerce, and industry for the entire southern island of the Philippines. Since 1991, the responsibility for delivering basic health services, including family planning, has been decentralized to the local government units—provinces, cities, and municipalities—with a funding mix from local and national sources. However, despite decades of family planning initiatives in the country, implementation of these initiatives in Davao City still heavily relies on support from the regional office of the Department of Health (DOH) for commodities and training.

Despite ongoing efforts to promote gender equity in family planning, an analysis of service data indicates a significant gender disparity in utilization rates. A persistent trend of limited male involvement in family planning has long been evident in the years before the COVID-19 pandemic, as observed in 2019 when, of the 158,841 current users of family planning users in Davao City, 94.4% were female and only 5.6% were male users (unpublished data). This gender imbalance places a disproportionate health burden on women, leaving them increasingly vulnerable to health risks, side effects, and the psychological stress of managing contraception alone. The lack of shared responsibility in family planning also heightens the potential for women to face challenges in negotiating contraceptive use, making them more susceptible to unintended pregnancies and the associated physical, emotional, and economic consequences. In response, the Davao City Health Office (CHO), through its Population Division, launched a flagship program centered on NSV to address the gap.

### Program Development and Implementation

Davao City’s Family Planning for Men Program Through No-Scalpel Vasectomy (NSV Program) was officially started in 2008 by a group of male doctors at the CHO to increase male participation in family planning. Initially, NSV was administered as an in-office procedure at the CHO, but as the program gained traction, it evolved into a specialized service provided at the Davao City Vasectomy Center, a dedicated facility established to provide this procedure and men’s health services. NSV, which usually costs PhP 14,000 (US$250) in private hospitals, is provided for free at the city’s health facilities.

**FIGURE 1:**
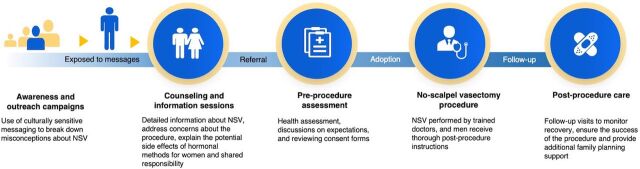
Cascade of Services in the No-Scalpel Vasectomy Program, Davao City, Philippines Abbreviation: NSV, no-scalpel vasectomy.

Since its launch, the NSV Program has served as a platform for educating and empowering men to take an active role in family planning decisions, positioning them as indispensable stakeholders in promoting the well-being of their families. The key program activities include conducting regular information drives and Family Planning for Men information campaigns at the community level using both traditional platforms (i.e., radio) and social media to dispel misconceptions surrounding family planning methods, particularly NSV. The outreach efforts aimed to raise awareness of all family planning options, ensuring that participants were aware of alternative methods before making a choice. To uphold the principles of voluntarism and informed choice, counseling sessions emphasized the non-coercive nature of the procedure, allowing men to make fully informed decisions about their reproductive health. Additionally, the NSV Program promotes male attendance at prenatal visits and family planning counseling sessions with their partners. The Family Planning for Men initiative is part of a comprehensive family planning campaign that focuses on 4 fundamental principles: (1) birth spacing, allowing mothers’ bodies to recover between pregnancies, reducing health risks and complications; (2) commitment to life, aiming to minimize maternal and infant deaths during childbirth; (3) informed choice, providing comprehensive information on the various contraceptive methods to enable couples to make educated decisions; and (4) planned parenthood, encouraging responsible family planning choices. The NSV Program’s expansion over the years reflects the strong support from the local government, which actively advocates for the program and champions family planning as a vital public health initiative.^16^ Today, the Davao City Vasectomy Center stands as the primary hub for NSV services, integrating the program into a broader, more accessible framework for men’s family planning (Figure 1).

The NSV Program’s expansion over the years reflects the strong support from the local government, which actively advocates for the program and champions family planning as a vital public health initiative.

### Partnership and Program Sustainability

The CHO’s commitment to the NSV Program extends to fostering strong partnerships with the private sector. In addition to coordinating program implementation with the regional DOH, the city has forged collaborations with organizations like DKT International, Cooperative Movement for Encouraging NSV, and the U.S. Agency for International Development. These partnerships provide crucial support, including training, logistical assistance, and technical guidance.

The shared values underlying these partnerships center on a mutual commitment to improving family planning access and outcomes. For private organizations, collaborating with local governments presents an opportunity to align their missions with public health objectives while also boosting brand visibility and credibility. Development partners can contribute their technical expertise and support government policies aimed at advancing reproductive health. For example, the ReachHealth Project, a 6-year activity funded by the U.S. Agency for International Development and implemented by RTI International, supported demand generation activities, including social and behavior change campaigns; assisted in routine service provision and outreach activities; and facilitated training to additional service providers. Additionally, the Project donated a MadaJet Anesthesia Device Kit, enabling painless NSV procedures by removing the need for scrotal injections. The use of the device not only enhances the overall client experience but also addresses a common concern among men regarding the procedure by alleviating fears associated with traditional anesthetic methods. The Project also strengthened the capacity of the Davao City Vasectomy Center to serve as a training hub for service providers from other cities and provinces. It further assisted in obtaining certification from the DOH, officially accrediting the facility as a training institution for NSV.

### Program Growth and Impact

**FIGURE 2:**
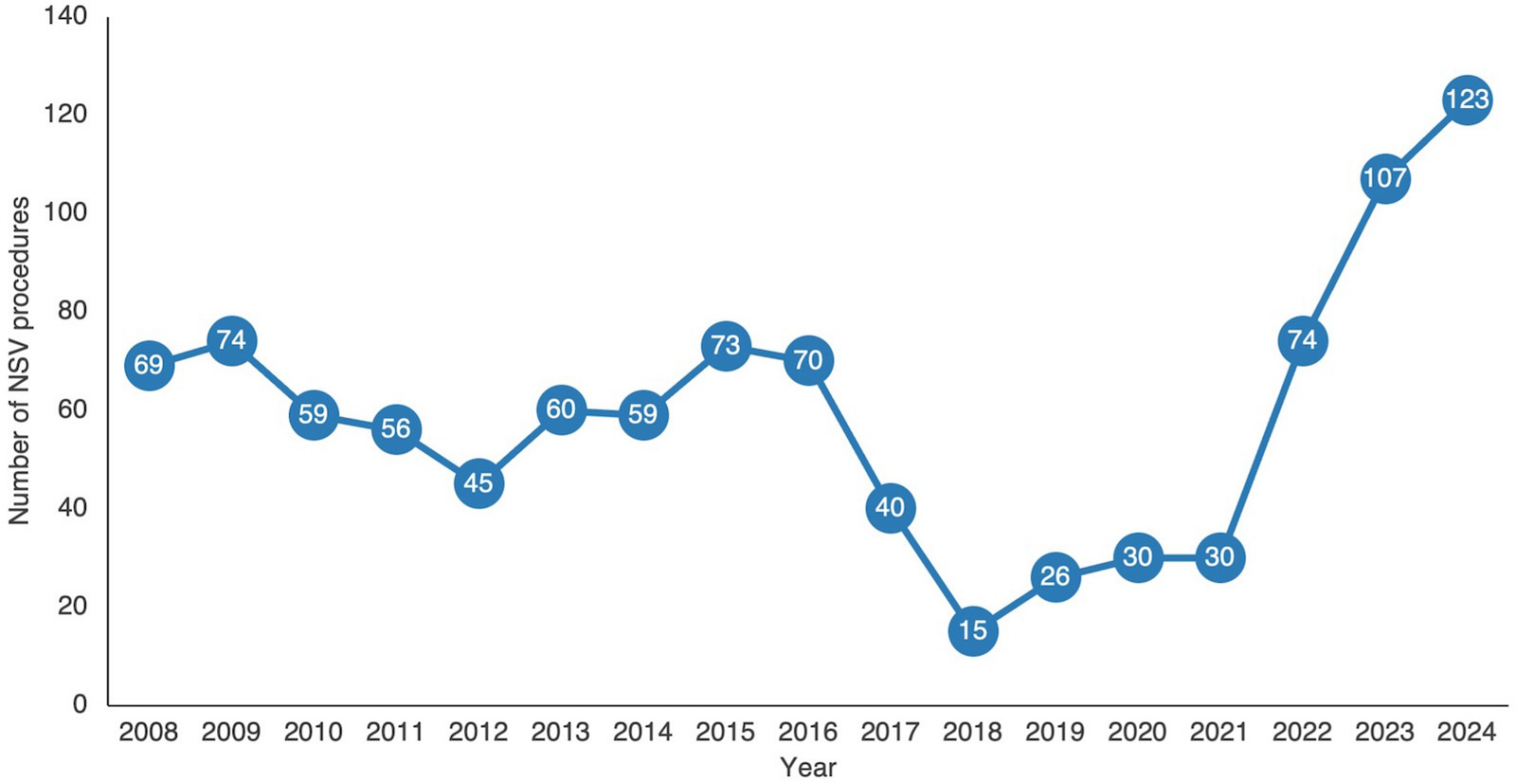
No-Scalpel Vasectomy Services Provided, Davao City, Philippines, 2008–2024 Abbreviation: NSV, no-scalpel vasectomy.

Since 2008, the program has consistently yielded promising results, with an average of 55 NSV procedures performed annually. Although the numbers dipped during the COVID-19 pandemic, the last 3 years have seen a resurgence with a remarkable 80% increase in procedures—rising from a pre-pandemic average of 56 to 101 procedures per year. While still modest, this surge reflects a growing acceptance of NSV among men in Davao City, affirming its appeal as a viable family planning option for couples. Importantly, the program operated without any targets or quotas, ensuring that the focus remained on promoting informed choices rather than merely achieving numerical goals. Sustaining these numbers over time is vital, as it reflects consistent engagement and gradual cultural shifts. As a result, Davao City is emerging as a pioneering force in promoting men’s involvement in family planning across the country. The program has not only attracted clients from within Davao City and its neighboring areas but also from other main island groups in the Philippines (Figure 2).

The NSV Program presents a compelling intervention model that can potentially instigate positive transformation in family planning gender dynamics, both locally and on a broader scale. The documented progress, fueled by local commitment and strong public-private partnerships, sets a remarkable precedent for future reproductive health initiatives and, as such, offers valuable insights for guiding program interventions and policy development in the Philippines and similar settings.

## CHALLENGES AND INSIGHTS

The NSV Program in Davao City has not been without its share of development and programmatic challenges. We highlight the following key challenges as well as the insights gathered from the implementation experience.

### Lack of Male-Friendly Facilities for No-Scalpel Vasectomy Presents a Barrier to Access

During the early stage of program implementation, securing suitable facilities or spaces for conducting NSV procedures posed a considerable challenge. Previous studies already noted that the absence of dedicated service delivery points impeded access to these services, rendering them less “male friendly” (e.g., predominance of female providers).^17^^,^^18^ This further reinforces the perception among men that family planning is a woman’s activity. To address this issue, the promotion of the city’s health centers and district hospitals as dependable and safe venues for men seeking family planning services was prioritized. Subsequently, the establishment of the Davao City Vasectomy Center proved to be a crucial step in overcoming this challenge, reflecting the importance of integrated service environments that encourage greater male participation in family planning, as supported by previous studies on improving access and uptake through male-friendly facilities.^19^

### Deep-Rooted Cultural Associations Between Masculinity and Fertility Affected No-Scalpel Vasectomy Uptake

Prevailing beliefs regarding masculinity, fertility, and family dynamics have fueled misconceptions about male sterilization,^19^^,^^20^ often perceived as emasculating and contradictory to traditional notions of manhood. Men, influenced by these cultural norms, may have a strong reluctance to undergo vasectomy due to fears of adverse effects on sexual performance and health.^17^^,^^19^ Recognizing and addressing these misconceptions was important for the NSV Program, as the societal expectation on men to conform to stereotypical “macho” demeanor is pervasive as well in the Philippine context.

To tackle this challenge, the CHO adopted culturally sensitive strategies to debunk the myths surrounding NSV and emphasized the importance of shared decision-making between couples. Key approaches included advocating for vasectomy among existing clients to serve as champions of the procedure and raising awareness of the side effects women experience when using hormonal methods. Information campaigns like the FP in Men, along with involving men in counseling sessions, further helped them to be more supportive of contraceptive use.

### Budgetary Limitations Necessitated Efforts to Secure External Resources

Despite the local government support, insufficient financing presented another hurdle, even with the local government units’ commitment to the program’s objectives. The administrative and logistical needs of the program necessitated a strategic plan to secure long-term funding to operate it sustainably. The CHO responded with a thoughtful and politically informed approach, seeking external expertise and resources through public-private partnerships. Running the program did not only test Davao City’s efforts to counter patriarchal norms but also resilience in the face of developmental and programmatic challenges. Since 2008, the Davao City Vasectomy Center has served 1,010 clients as of March 2024 and is the only long-standing NSV implementer in the Philippines offering it for free. This adaptability resulted in the program’s growing recognition and attracting clients from areas beyond Davao City.

## CONCLUSION

The NSV Program in Davao City serves as a promising model for engaging men in family planning and addressing gender disparities in such initiatives. By adopting culturally sensitive approaches, sustainable funding strategies, and private sector partnerships, the program has effectively addressed infrastructure challenges and dispelled cultural misconceptions. With strong local support, these strategies hold the potential to sustainably increase male participation in family planning and enhance reproductive health outcomes within communities.

## Notes

**Peer Reviewed**

**First Published Online:** October 22, 2024.
